# Blood management in hip fractures; are we leaving it too late? A retrospective observational study

**DOI:** 10.1186/s12877-019-1099-x

**Published:** 2019-03-12

**Authors:** Gillian Puckeridge, Morne Terblanche, Marianne Wallis, Yoke Lin Fung

**Affiliations:** 1 Department of Orthopaedics, Sunshine Coast Hospital and Health Service, Birtinya, Queensland 4575 Australia; 2 School of Health and Sports Science, University of the Sunshine Coast, Sippy Downs, Queensland 4556 Australia; 3 Safety Quality and Innovation, Department of Anaesthesia and Perioperative Medicine, Sunshine Coast Hospital and Health Service, Birtinya, Queensland 4575 Australia; 4 School of Nursing, Midwifery and Paramedical Science, University of the Sunshine Coast, Sippy Downs, Queensland 4556 Australia

**Keywords:** Hip fracture, Bleeding, Anaemia, Blood Transfusion

## Abstract

**Background:**

Anaemia in hip fracture patients has been associated with increased risk of allogenic blood transfusion (ABT), poorer functional outcomes and increased mortality. Few studies have reported the prevalence of anaemia on admission or its progression prior to surgery in this cohort. We aimed to measure the prevalence of anaemia on admission in older persons who sustain a hip fracture, identify if anaemia develops or progresses prior to surgery, and to report associations with outcome.

**Methods:**

A retrospective, observational study was undertaken in a regional hospital. All patients aged 60 and over, admitted with a primary hip fracture resulting from a simple fall, in the 12 months of 2014 were included. The World Health Organization (WHO) definition of anaemia was used. Pathology databases and clinical records were reviewed to collect data. Repeated measures ANOVA’s were used to quantify the progression of anaemia prior to surgery, and Chi square test were used to report associations with outcome variables.

**Results:**

Two hundred sixty-one patients were identified, median age was 81 years. There were twice as many females as males and just over half the sample had extracapsular fractures. Anaemia was present on admission in 45% (*n* = 117), highest incidence of anaemia occurred in males 52.0% (*n* = 39), extracapsular fractures 41.9% (*n* = 78) and those aged over 80 years 49.7% (*n* = 91). Progression of anaemia prior to surgery was significant in all groups (*p* < 0.05), with the greatest reduction seen in extracapsular fractures. Pre-surgery reduction in Hb was recorded in 82.3% of patients between admission and day 1, and in 71.4% between admission and day 2. There was significant association between anaemia on admission and PRBC transfusion (*p* < 0.05), in hospital mortality (*p* < 0.05) however no association with the use of antiplatelet or anticoagulant medication, nor LOS.

**Conclusions:**

The findings demonstrate that pre-surgical anaemia in older hip fracture patients is associated with a PRBC transfusion and increased hospital mortality. Importantly, it also identified that patients continue to bleed after admission, leading to the development of or worsening anaemia. Thus, identification anaemia in the pre-surgical period provides an opportunity for treatment to avoid transfusions and improve patient outcomes.

## Background

Hip fractures in the elderly are associated with poor outcomes. The comorbidity of these older patients combined with the physiological stress of injury and surgery contribute to poorer outcomes [1, 2]. One physiological marker that contributes to poor outcomes in hip fracture patients is anaemia due to low haemoglobin (Hb). Anaemia in older surgical patients is associated with increased mortality and length of hospital stay (LOS), and poorer functional outcomes [3] . In addition, low admission Hb levels are also a predictor of transfusion risk in hip fracture patients [4, 5]. Internationally, more than 42% of hip fracture surgery patients present with anaemia on admission [6, 7] however this has not been investigated in Australia.

Anemia in elderly patients with hip fracture has been traditionally attributed to surgical or post-surgical bleeding [8]. While there is a growing acknowledgement of the importance of pre-operative detection of anaemia in surgical patients [9], there is little guidance in the emergency setting and in the elderly hip fracture cohort. Data is lacking as to whether these patients continue to bleed between admission and surgery and what effect this may have on patient outcomes. In this study we sought first, to measure the prevalence of anaemia on admission in elderly people who sustain a hip fracture and second, identify if anaemia progresses prior to surgery. If anaemia is present on admission and worsens prior to surgery, this will confirm that the period before surgery is a crucial period for anaemia identification and management. We also sought to investigate whether there was any association between anaemia on admission, with the use of antiplatelet or anticoagulant medications, transfusion of packed red blood cells (PRBC), increased LOS and in hospital mortality.

## Methods

This retrospective, observational study was undertaken in a Queensland regional hospital servicing a population of approximately 390,000 people. Ethical (HREC/15/QPCH/55) and Queensland Public Health Act approvals were granted for this study. Consent to participate was not applicable as this was a retrospective study and access to the patient level information without consent was granted through the Public Health Act approval. All patients aged 60 years and over, admitted with a hip fracture resulting from a fall from no greater than standing height, in the 12 months of 2014 were included in the study. Following the exclusion of pathological and peri-prosthetic fractures, 261 patients were included in the sample.

### Data collection

The World Health Organization (WHO) defines anaemia as a Hb level of less than < 13 g/dL in men and < 12 g/dL in women [10]. Data were abstracted from pathology databases and clinical records were audited to collect the following inpatient data between admission and discharge: demographics (age and sex), fracture type (intracapsular or extracapsular), use of anticoagulant or antiplatelet medication (excluding aspirin), serial Hb pre-surgery (as available), LOS, administration of PBRC during the admission and in hospital mortality.

### Statistical analysis

The data were checked for completeness and variables for the normality of their distribution. Statistical analysis was completed with software package SPSS (V 23.0. SPSS, Chicago). Descriptive statistics for the data are reported as frequencies and percentages or measures of central tendency and distribution as appropriate to the level of measurement and normality of the data. Repeated measures analysis of variance (ANOVA) was used to measure the progression of preoperative anaemia. Associations between anaemia and antiplatelet / anticoagulant medication, transfusion of PRBC, LOS and in-hospital mortality were conducted with Chi square tests, as appropriate. *P* values less than 0.05 were considered significant.

## Results

A total of 261 patients with primary hip fracture were identified. The median (IQR) age was 81 (11) years and 70.1% of the sample were aged 80 years or over (Table 1). There were twice as many females (*n* = 186) as males (*n* = 75) and just over half the sample had extracapsular fractures (*n* = 140) (Table 1). Mean time to surgery, time to postoperative transfusion and median LOS were similar in both groups (Table 1). Overall, in hospital mortality was 3.8% with little variation between genders.

**Table 1:** Cohort demographics comparing males and females

	Male	Female	Total
n (%)	75 (28.7)	186 (71.3)	261 (100)
Age median (IQR)	83 (13)	84 (10)	81 (11)
IC # n (%)	33 (44.0)	88 (47.3)	121 (46.4)
EC # n (%)	42 (56.0)	98 (52.6)	140 (53.6)
Time to surgery mean (SD)	1.27 (0.96)	1.20 (0.77)	1.23 (0.83)
Anticoagulant/Antiplatelet Medication (%)	19 (25.3)	43 (23.1)	62 (23.7)
LOS median (IQR)	7 (6)	6 (5)	5 (6)
In hospital mortality	3 (1.1)	7 (2.7)	10 (3.8)

Legend: *IC* # Intracapsular fracture, *EC* # extracapsular fracture, *LOS* length of stay

Mean Hb levels, the percentage of the cohort with anaemia, the percentage with a reduction in Hb in the pre-operative period and post-operative blood transfusion are reported, for each sub-group, in Table 2. Anaemia was present on admission (D0) in 44.8% of patients (*n* = 117), with a mean (SD) admission Hb of 12.3 g/dL (1.58), for the whole sample. The incidence of anaemia on admission was higher in males (52.0%) than females (41.9%). Notably there was a higher incidence of anaemia on admission with extracapsular fractures (53.2%) and those aged over 80 years (49.7%).

**Table 2:** Mean (SD) of Hb levels and percentages of patients by subgroup who had anaemia, percentage reduction in Hb and significance or reduction over time and postoperative blood transfusion percentage

Groups	Patients n (%)	Hb g/dL mean ± SD			Anaemia by subgroup n(%)			Reduction in Hb by subgroup (n)%		Postoperative transfusion of PRBC n (% sample)
		DO (*n* = 261)	D1 (*n* = 175)	D2 (*n* = 51)	D0	D1	D2	D0-D1	D1-D2	
All	261 (100)	12.3 (1.57)	11.4^a^ (1.68)	11.1^b^ (1.81)	116 (44.8)	88 (50.9)	33 (69.4)	143 (82.3)	34 (71.4)	75 (28.7)
Male	75 (28.7)	12.5 (1.77)	11.8^a^ (2.02)	11.7^b^ (1.94)	39 (52.0)	33 (69.3)	12 (75.0)	(36) 73.5	12 (78.6)	18 (6.9)
Female	186 (71.3)	12.2 (1.49)	11.3^a^ (1.52)	10.7 (1.68)	77 (41.9)	85 (68.3)	21 (66.7)	107 (85.7)	(21) 67.9	58 (22.2)
Intracapsular Fracture	121 (46.4)	12.7 (1.49)	12.1^a^ (1.55)	11.8^b^ (1.89)	41 (34.7)	41 (49.4)	12 (48.0)	63 (75.3)	(20) 80.0	15 (5.7)
Extracapsular Fracture	140 (53.6)	12.0 (1.56)	10.8^a^ (1.53)	10.2 (1.31)	74 (53.2)	77 (85.6)	21 (92.0)	80 (88.9)	14 (63.6)	61 (23.4)
60–79 years	78 (29.9)	12.7 (1.59)	12.0^a^ (1.77)	12.2 (1.46)	25 (32.9)	26 (52.9)	5 (45.5)	41 (80.4)	7 (66.7)	17 (6.5)
≥ 80 years	183 (70.1)	12.1 (1.54)	11.2^a^ (1.59)	10.7^b^ (1.78)	90 (49.7)	91 (74.2)	28 (76.3)	102 (83.1)	26 (72.7)	59 (22.6)

Legend: *Hb* haemoglobin; anaemia = Hb < 13 g/dL in males and 12 g/dL in females, *PRBC* packed red blood cells, *D0* day of admission, *D1* day after admission (pre-operative), *D2* second day after admission (pre-operative); D0 – D1 ^a^ = *p* < 0.05; D1 – D2 ^b^ *p* < 0.05

There was a significant reduction in Hb between admission and day 1 pre-surgery, with a mean reduction of 0.9 g/dL (Wilks’ Lambda = 0.538, F (1,178) = 148, *p* = 0.000) (Table 2). In this period, 82.3% of the cohort experienced a reduction in Hb and this occurred irrespective of gender, type of fracture or age. Of the patients who had still not gone to surgery on day 2, 71.4% of patients recorded a Hb reduction that averaged 1.2 g/dL relative to admission Hb, (Wilks’ Lambda = 0.693, F (1,40) = 18, p = 0.000). On day 2 pre-surgery significant Hb reductions were only recorded with males, intracapsular fracture and in patients aged 80 years or more. As a result of the decrease in Hb between D0 and D1, the proportion of patients defined as anaemic increased in all groups irrespective of gender, type of fracture or age. The biggest increase in proportion of patients with anaemia was with extracapsular fractures and patients aged ≥80 years. On the second day pre-surgery there was a further increase in the proportion of male patients (75%) and those with extracapsular fractures (92%) who met the criteria for anaemia.

The overall the postoperative transfusion rate was 28.7%. The highest rate occurred in extracapsular fractures (23.4%) and those aged 80 or over (22.6%). Transfusion of PRBC occurred in 40.4% (*n* = 47) of patients who were anaemic on admission and only 19.3% (28 patients who were not anaemic on admission. There was a statistically significant association between anaemia on admission and day 1 pre-surgery with PRBC transfusion (*x*^*1*^ = 15.31, *df* = 2, *p* = 0.000) but not with anaemia on day 2 pre-surgery. Hospital mortality was similar between males and females, and was associated with on-admission anaemia in the whole cohort (*x*^*1*^ = 7.46, *df* = 2, *p* = 0.006), however not anaemia on day 1 or day 2 pre-surgery (see Table 3).

**Table 3:** Chi square association between anaemia, transfusion and in hospital mortality

	n (% of sample)	*X*^*1*^ (*df)*	*p* value
Postoperative transfusion of PRBC			
Admission	47 (40.5)	15.15 (2)	0.00
Day 1 pre surgery	43 (36.1)	18.63 (2)	0.00
Day 2 pre surgery	10 (30.3)	3.38^a^	0.19
In-hospital mortality			
Admission	8 (6.8)	7.54 (2)	0.07
Day 1 pre surgery	7 (5.8)	3.76^a^	0.12
Day 2 pre surgery	1 (3.0)	0.09^a^	1.00

^a^ Fishers exact test

The highest hospital mortality was seen in those who presented with anaemia (6.9%) and patients aged 80 or over (4.4%). There was no statistically significant association between the presence of anaemia on admission, the use of antiplatelet (clopidogrel *n* = 24) or anticoagulant (warfarin *n* = 38) medication (*x*^*1*^ = 2.40, *df* = 2, *p* = 0.30) or LOS (*x*^*1*^ = 27.66, *df* = 27, *p* = 0.43). The time to surgery for patients on these medications was not statistically significantly different.

## Discussion

In this study, 44.8% of older patients with a hip fracture were anaemic on admission and the proportion of patients with anaemia increased between admission and surgery. Reductions in Hb between admission and surgery was associated with post-operative transfusion of PRBC and in-hospital mortality. While anaemia in elderly hip fracture patients is well documented during the operative and post-operative period [11, 12], data from this study has shown that these patients actually continue to bleed in the first 24–48 h following their fall, as they wait to go to surgery.

The percentage of patients, who experienced anaemia on admission in this study, is similar to other reports [13, 14]. In other studies, the measurement of Hb has only been reported in cohorts limited by fracture type or a time to surgery greater than 4 days. This study included all elderly (aged 60 years or more) hip fracture patients regardless of fracture type or time to surgery. Our data show irrespective of age and fracture type that greater than 30% of elderly patients are anaemic on admission. This suggests that on admission anaemia assessment would be valuable in all hip fracture patients.

Progression of anaemia as measured by Hb occurred in all groups particularly in the first 24 h following admission. Although this has previously been reported [13, 14], this is the first time that data have been analysed in all elderly hip fracture patients regardless of fracture type, gender, age and time to surgery. As 23.7% of the patients were on clopidogrel or warfarin we questioned if these medications could have contributed to anaemia, but no statistically significant association was detected. The small number of patients prescribed anticoagulants precluded analysis by drug class. By age stratifying the cohort we have shown that a higher proportion of patients aged 80 years or older became anaemic prior to surgery, than their younger counterparts. This data identifies patients aged 80 years or older as a sub group that are at higher risk of pre-surgical anaemia.

Similar to other studies, [13, 14] patients with extracapsular fractures, and female patients had the greatest progression of anaemia between admission and day one pre-surgery. Interestingly, males were more anaemic than females on admission and their anaemia worsened as they waited for surgery, This gender difference in Hb of elderly adults is not unique as some studies have shown that while Hb levels of females increase in middle age, the Hb of males decreases with age [15]. However, this gender difference is significant as a prospective cohort study of 6880 aged (65 year or more) adults found that even mild anaemia is an independent risk factor for a nearly two-fold increase in 5-year all-cause mortality for apparently healthy men [16].

By age stratifying the cohort we have shown that a higher proportion of patients aged 80 years or older became anaemic prior to surgery, than their younger counterparts. These data identify patients aged 80 years or older as a sub group that is at higher risk of pre-surgical anaemia.

There are reports that show early surgical intervention in hip fracture is associated with improved outcomes [17, 18]. Our finding that the greatest reduction in Hb was seen in the first 24 h following admission, emphasises the need for early anaemia assessment to prevent further reductions in Hb and adds support to early surgical intervention, and consequently improved patient outcome. In settings where there are resource limitations, delays to hospital presentation and delays to surgery, early and daily anaemia assessment through commonly available Hb measurement and a review of any anticoagulant use could be considered. There are a number of interventions including iron therapy, antifibrinolytic agents and rHuEPO-based treatment that have proven effective in reducing bleeding and reversing anaemia in surgical patients [9], but these have yet to be trialled in the pre-surgical elderly hip fracture patient. Future consideration should be given to the investigation of the transferability of these interventions such as anti-fibrinolytic agents and pre-surgical iron formulations in the emergent pre-surgical hip fracture cohort.

**Fig. 1:**
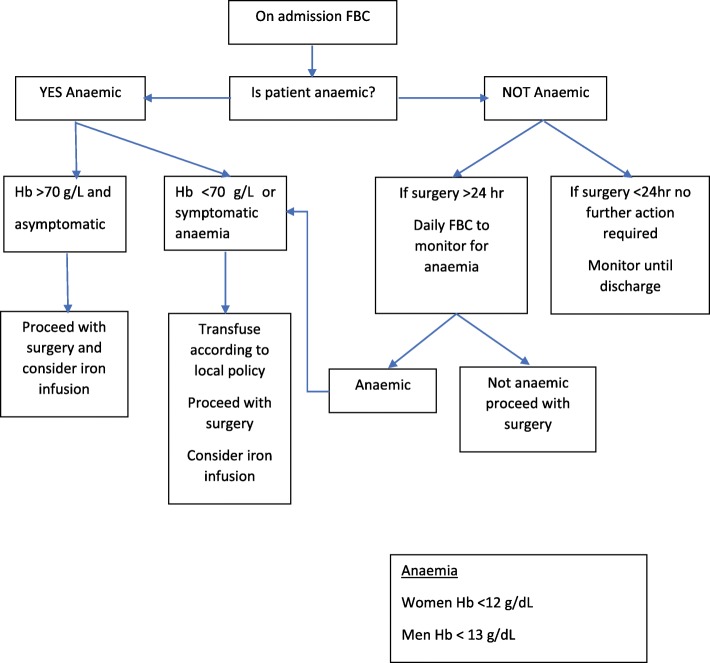
Proposed Preoperative Anaemia Assessment and Treatment Pathway

In this study, anaemia on admission was associated with post-operative transfusion of PRBC and in-hospital mortality, but not LOS or the use of anticoagulants. We did not assess transfusion thresholds based on Hb levels because we employ a single unit transfusion policy based on clinical assessment. Whether the small sample size of this study contributed to the lack of association with LOS is unclear. The small number of patients prescribed anticoagulants precluded analysis by drug class. Other studies based on post-operative data identify an association between anaemia on admission, post-operative transfusion of PRBC and in hospital mortality [11, 19]. Where surgical delay cannot be avoided, we propose that anaemia assessment is a simple evidence based pathway to identify when interventions to prevent or correct anaemia are required (Fig. 1) and future studies to test such a pathway should be undertaken.

Limitations of this study include the retrospective design of the study. This precluded the measurement of all potential outcomes, confounding factors like comorbid diseases, infection, peri-operative complications and serial Hb measurements on all patients. Though the sample size is small, the study provides useful preliminary data to substantiate further studies.

Results from this study point to the need for future investigation into modifiable factors associated with pre-surgical bleeding in hip fractures such as coagulation derangement, the influence of haemodilution on bleeding and the progression of anaemia. While there are clear guidelines regarding anaemia correction in most surgical cohorts, it is unclear in the geriatric hip fracture cohort if this should occur prior to, during or after surgery. The timeliness of anaemia correction and effect on outcomes is something worthy of future examination. There is also a need for robust randomised clinical trials investigating the effect of pre-operative interventions aimed at reducing bleeding and reversing anaemia.

## Conclusion

Data from this study demonstrate that elderly hip fracture patients continue to bleed after admission, and that preoperative anaemia is clearly associated with a higher PRBC transfusion rate and higher mortality. Hence, the period between admission and surgery provides a valuable window of opportunity to correct anaemia. Early pre-surgical identification of anaemia is imperative in this frail cohort to ensure timely and appropriate interventions to treat the anaemia, and to improve surgical outcomes.

## Abbreviations

ABT: Allogenic blood transfusion
Hb: Haemoglobin
IHM: In hospital mortality
LOS: Length of stay
PRBC: Packed red blood cells
WHO: World Health Organisation
